# Supplementary material for: The choice of Madrid as the capital of Spain by Philip II in the light of the knowledge of his time: A transport network perspective
Source: PLoS One. 2022 Jun 15;17(6):e0269419. doi: 10.1371/journal.pone.0269419 (PMC9200313; doi:10.1371/journal.pone.0269419)
Supplement: S1 Annex — (DOCX) [file pone.0269419.s002.docx]

**Annex 1**

To determine the differences in costs between the two means of transport, the centrality of intermediation and the accessibility of the main towns and villages were calculated using as network an enlarged transport map (Fig A1) to which the maritime connections between the main ports have been added to the roads shown in Fig 10.

The included ports correspond to those that appear as the most important ones in the Wit’s map titled *Totius Europae littora novissimè edita* [171].


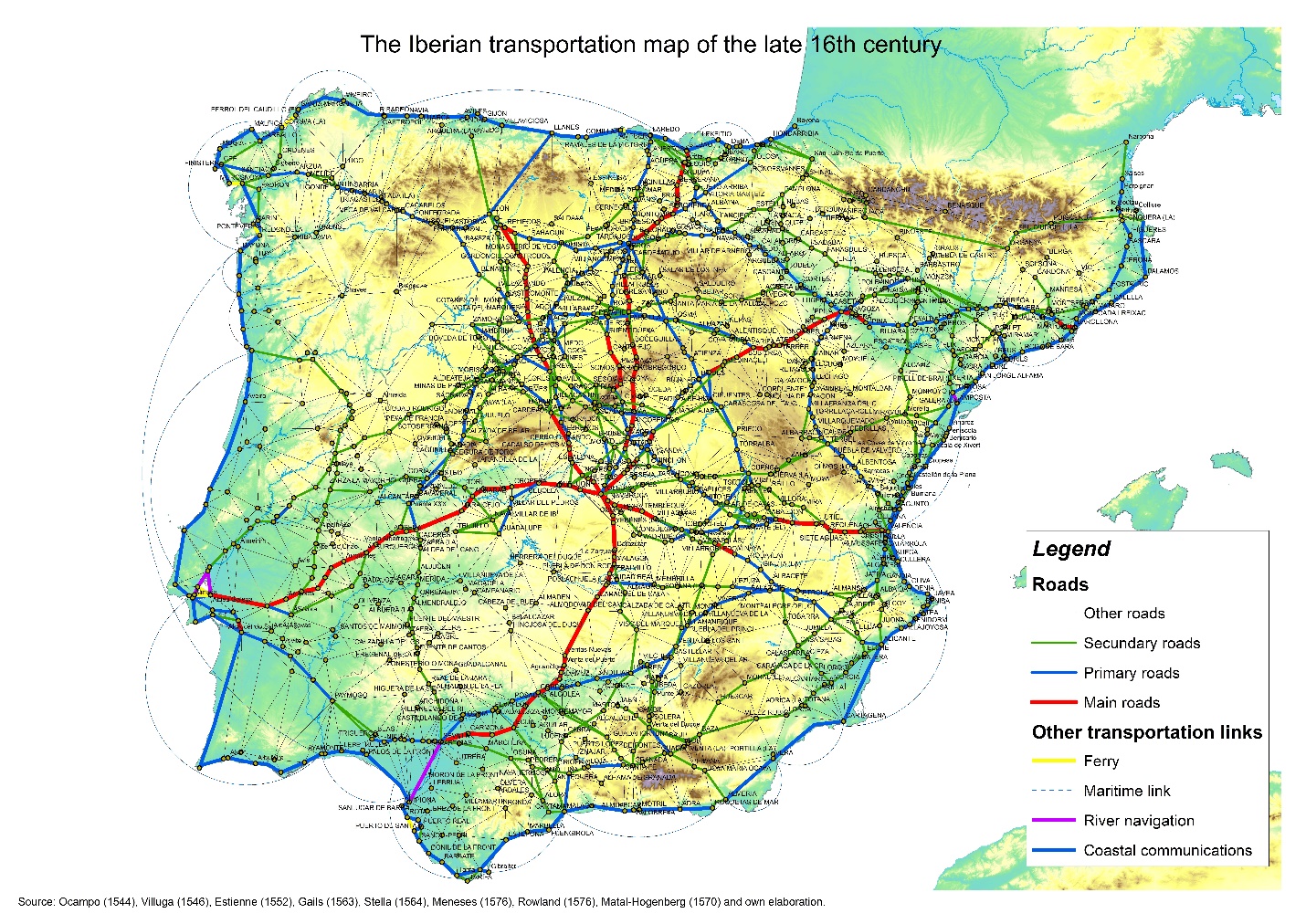


**Fig A1. The Iberian transport map of the late 16th century** Source: Own elaboration. Base map from the Centro Nacional de Información Geográfica (Spain).

The results obtained using different maritime-land transport cost ratios (Figs A2 and A3) were compared with the map elaborated by Menéndez Pidal [18] in which the roads are categorized according to the frequency in which they are cited, assuming that the sections that appear repeated on the most occasions are also the most important or frequented [149]. Additionally, they were compared with those obtained by the authors [101] for the 18^th^ century under the commonly accepted assumption that there were no significant changes in the road network between 1550 and 1700 [67].


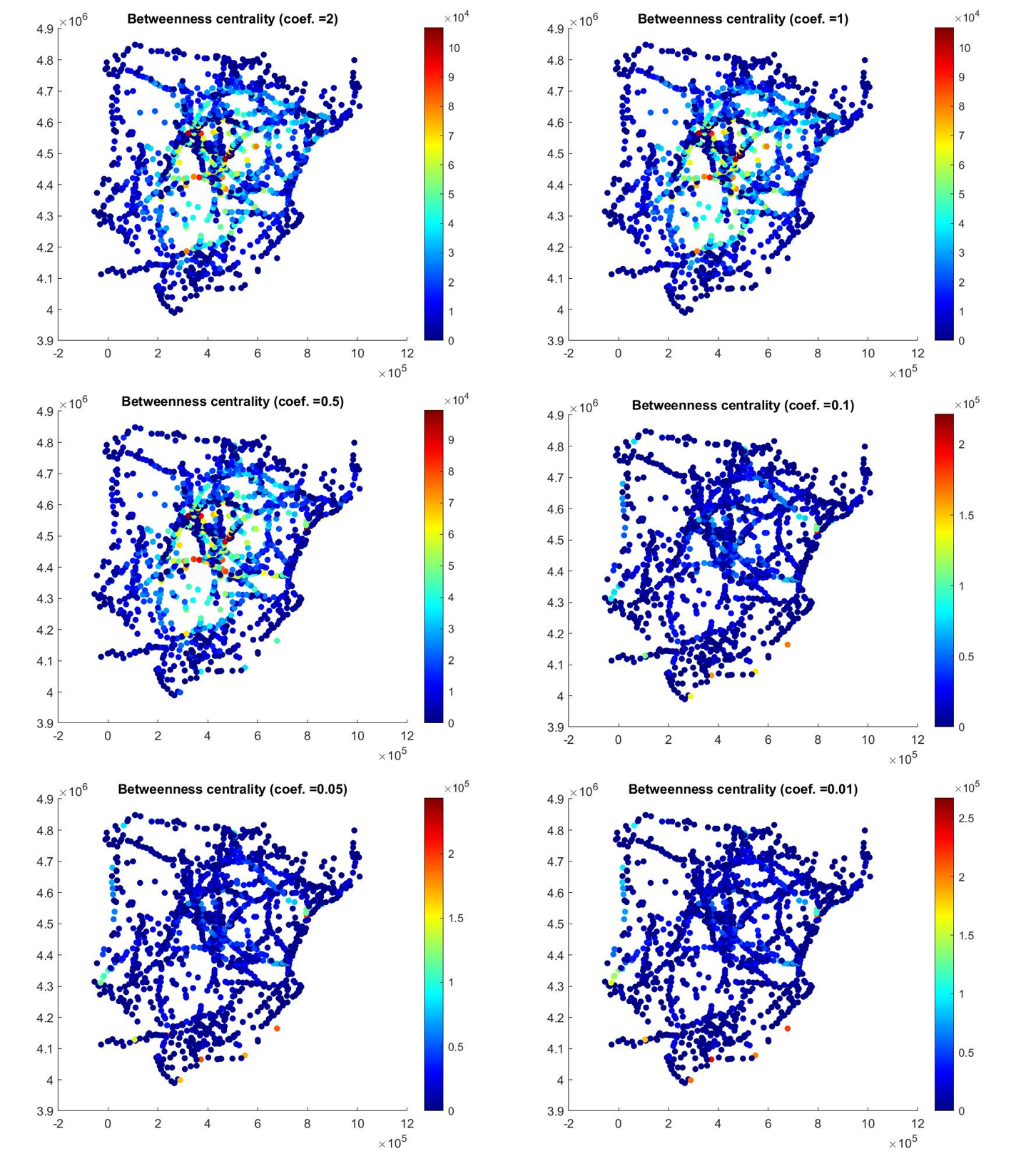


**Fig A2. Betweenness centrality and relative advantages of maritime transport over land transport.** Source: Own elaboration.

Maritime-land transport cost ratios lower than 1 generate traffic flows inconsistent with the observed road network, which indicates that the advantages of maritime transport in the pre-railroad era were much lower than those considered in Carreras and Soto [70] for Spain and closer to those of Munro [34] Ballaux and Blondé [35] for the Low Countries.


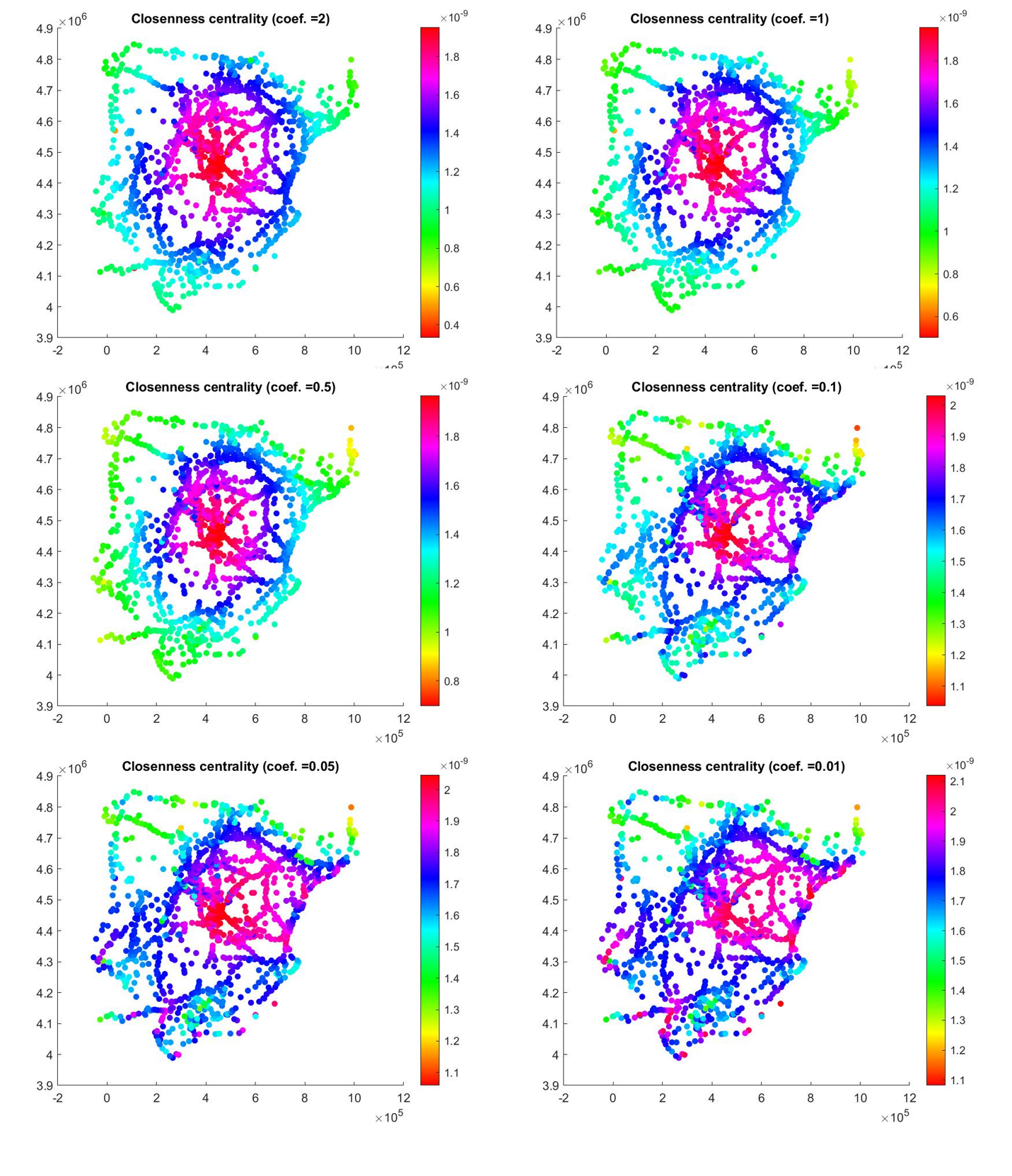


**Fig A3. Accessibility and relative advantages of maritime transport over land transport** Source: Own elaboration.

**Annex 2**

The routes listed in the anonymous map entitled *"* *Spagna con le distantie de li loci"* [122] show a limited degree of overlap with the itineraries of Villuga [63], indicating that essential roads are missing from this source.


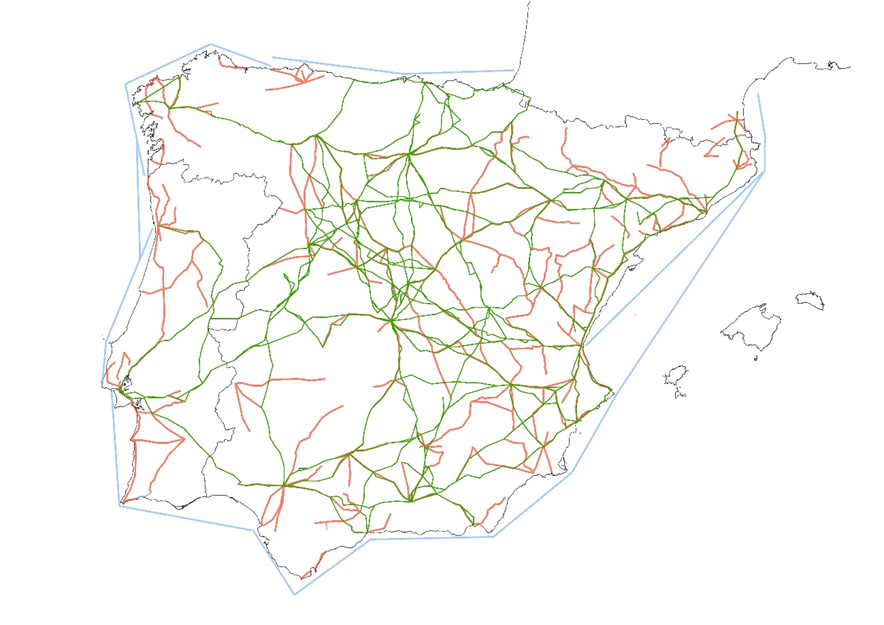


**Fig A4. Comparison of Villuga's itineraries with the routes shown on the map " Spagna con le distantie de li loci".** Source: Own elaboration. Base map from the Centro Nacional de Información Geográfica (Spain).

**Annex 3**

Although the lines that appear on *"* *Spagna con le distantie de li loci"* [122] do not precisely represent roads, but rather travel times between villages and should therefore be taken only as mere approximations, the significant degree of coincidence with the elaborated network confirms the soundness of the methodology used.


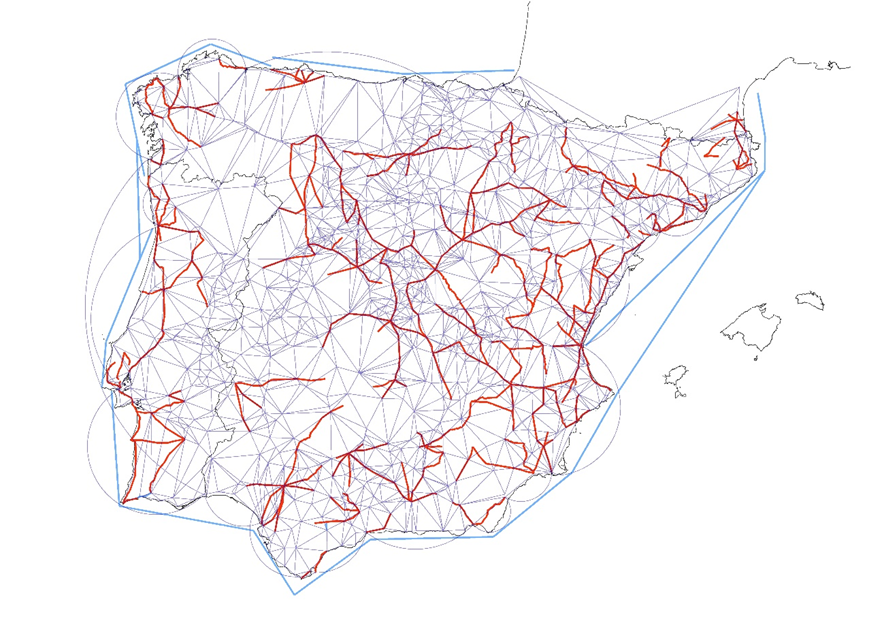


**Fig A5. Comparison of the routes shown on the map " Spagna con le distantie de li loci" with the road network developed.** Source: Own elaboration. Base map from the Centro Nacional de Información Geográfica (Spain).

In red and blue the routes of on *"* *Spagna con le distantie de li loci"*  [122], in grey the estimated road network.

**Annex 4**


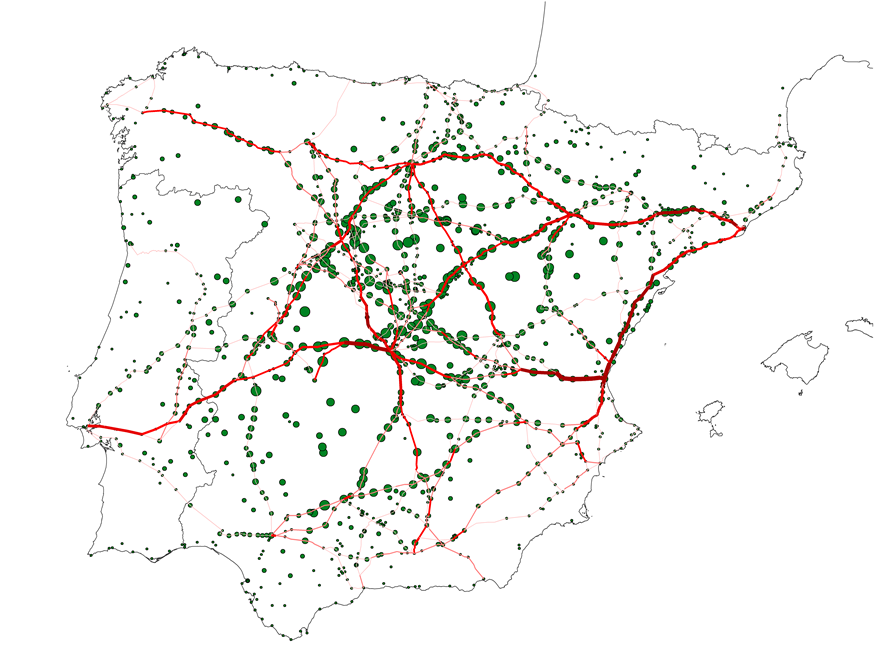


**Fig A6. Comparison of Villuga's itineraries with reconstructed road network (Betweenness centrality).** Source: Villuga [63] and own elaboration. Base map from the Centro Nacional de Información Geográfica (Spain).

**Annex 5**


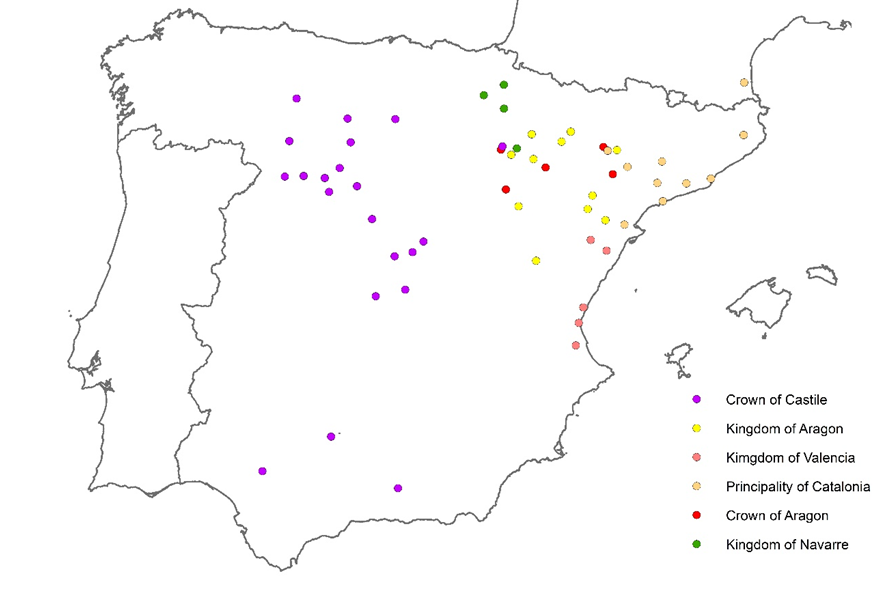


**Fig A7. Courts of the Spanish Kingdoms (13^th^ – 16^th^ centuries).** Source: Own elaboration. Base map from the Centro Nacional de Información Geográfica (Spain).
