# Supplementary material for: The choice of Madrid as the capital of Spain by Philip II in the light of the knowledge of his time: A transport network perspective
Source: PLoS One. 2022 Jun 15;17(6):e0269419. doi: 10.1371/journal.pone.0269419 (PMC9200313; doi:10.1371/journal.pone.0269419)
Supplement: Attachment — Submitted filename: Rebuttal letter.docx [file pone.0269419.s003.docx]

We would like to thank the editor and both reviewers for giving us the possibility to improve our work. We believe that the comments made are very constructive and appropriate. Their incidence on the expository weaknesses of the initial version of the article has not helped us to make a new one that we hope they consider substantially better.

We will now respond to the various criticisms and suggestions made.

**Reviewer #1:**

The paper provides an interesting look at the decision of establishing a permanent capital and court in Madrid by Phillip II. It is interesting to read how logistical costs could have influenced the decision at that time. The authors also do an outstanding effort in reconstructing and gathering the data available at that time.

Still, there are some areas that could be improved (overall flow of the manuscript principally) and some that could be added to increase the value of the paper (for example discussion of the importance of the results for other studies).

Thank you for your kind words. We have tried to improve the flow of the manuscript by clarifying the objectives and detailing the structure of the paper. We have included a brief final discussion on the importance of the research conducted for future work.

1. The **structure of the manuscript** could be made more explicit by improving the division in sections.

a. Just by looking at the main sections (introduction -> The intrinsic factors -> The network factors -> The Spanish road network -> Results -> Conclusion) could better guide the reader through the manuscript (which is particularly important considering the length)

The division into sections has been completely reworked. We feel that it is now better structured, and the section headings are more descriptive.

b. The introduction in its current form it is quite extensive. A shorter one would be preferable. As an example, “this paper aims to analyze Madrid’s suitability as Spain’s capital from an economic point of view” is in the seventh page.

We regret that the introduction is too long. The reason for this is that the work offers a quantitative view of the subject that clashes with the traditional view more focused on political conditioning factors. To facilitate the dissemination of the work in other academic sectors, we have tried to show that we have also paid attention to these issues.

c. In the page 9, the research is presented in several steps. However its clarity could be improved.

We have rewritten the description of the structure of the work that appears at the end of the introduction.

2. **Title** does not reflect the content

The title has been modified to better reflect the content of the paper. The new title is "The choice of Madrid as the capital of Spain by Philip II in the light of the knowledge of his time: a transport network perspective".

3. The **abstract** argues that “questioning the belief that this choice was eminently personal or political but lacked economic rationality”

a. Does the previous literature disregarded the economic merits of establishing the capital in Madrid?

There is a reference to Garcia Delgado (1990), which tells about

i. “unfavorable conditions for economic activity”
ii. “scarcity of primary resources”
iii. large distance to coastal trading centers and lack of fluvial traffic”. It seems that the paper focus mainly on the (iii), which is fine, but if that is the case, **it could be explicitly mentioned.**

Thanks for the comment. To the extent that no city has all the resources it needs the suitability of the location is largely determined by the cost of supplying them.

A paragraph has been introduced that refers to this issue.

b. What does economic rationality mean in the context of this paper?

It seems that the rationale is more focused on the logistical properties and how that could have influenced the final choice among the different possibilities. Please clarify it.

We start from the idea that it is not possible to know exactly the motivations that led Philip II to choose Madrid as capital. We only intend to show that his decision is not inconsistent with various economic criteria.

We have tried to clarify this question by means of a clearer wording of the text, especially in the final part of the work.

4. It is mentioned in different parts the comparison with other alternatives. The implicit understanding is that those would have been considered at that time (although it is not sure if that is based on the actual historical knowledge or it is the assumption from the authors, which is fine but should be explained).

a. Furthermore, the alternatives analyzed seem to differ throughout the text. For example, in the introduction “Besides Madrid, other cities have been pointed out as possible locations, such as Valladolid, Toledo, Seville, or even Barcelona or Lisbon”.
b. In Fig 1, other appear such as Malaga, Valencia, Murcia.

Thank you for your comment. To the best of our knowledge there was no definite list of alternatives. These are only conjectures about the options that Philip II is supposed to have been able to handle in his choice. The paragraph on page 5 referring to this question has been modified for clarification.

5. The paper employs several sources and methods to analyze each of the different variables. Whilst this is reasonable, it also adds some difficulty to its reading.

a. Page 9 “we analyze several Spanish cities’ suitability as potential capitals using a complex network approach”.
   i. Complex network approach could be further explained here

A brief explanation of the concept of complex networks has been included in the text along with some references that may be of interest to the reader interested in the subject.

6. The analysis of the three logistical costs (access to ports, fluvial traffic, and road transportation) could be followed by a comparison of these for all the cities considered (in reality or hypothetical based on the reasoning by the authors). It would be also interesting to include the potential differences in the weight of each in the final decision.

This suggestion would significantly improve the work, but the available information is very incomplete and does not allow for a detailed analysis by city, only a general overview. In any case, we will try to make progress on the subject in future works.

7. One concern that pop up in my mind through the text is why the logistical cost are estimated for the Spanish territory today and not others that were part of Phillip II reign. Please clarify.

As noted on page 5 of the paper, Philip II focused his interest on his peninsular dominions after being removed from the imperial crown by his father in 1531. Although he was king of Portugal from 1580, he was not king at the time he chose the capital. In any case, an analysis of all the European dominions was far beyond the scope of the paper. It would have been possible to include some small territories north of Catalonia consolidated after the peace of Cateau-Cambrésis in 1559. The only city of relative importance not included in the analysis is Perpignan. We considered that the inclusion in the analysis of this area meant an important extra work and the results would not be affected.

<https://i.redd.it/mfy9oketyli61.png>

8. It is not clear whether the paper looks to describe the decision-making process by Phillip II or to provide a general rationale that could have influenced any “rational” decision making.

a. If it is about Philipp II’s mainly, then the particularities and possible external influences on him could be included.
b. Or it could be to analyze how the logistical factors influenced his, based on the knowledge of the final decision.
c. Furthermore, it seems that all the factors considered had same weight (at least in logistics). Please clarify.

As noted in the answer to the third question posed, the intention was not to describe Philip II's personal decision-making process, but rather the rationale that a person with similar goals and knowledge might have followed. A sentence clarifying this point has been included on page 10.

9. There is also some information that could be easily included to facilitate the reading by non-experts on this specific issue

a. Context of the establishment of the permanent capital
   i. How it goes before? What was the historical context in which occurred?
   ii. The benefits and drawbacks of a permanent capital
   iii. What was the
   iv. These are somehow described in different parts, but it could be strengthened

A brief commentary on the issue has been introduced on page 1 along with some references that may be useful to the reader who wishes to go deeper into the subject.

b. A more structured description of the different “theories” of Phillip II, including also some of the aspects that could have influenced him (role of advisors, importance of the lessons from his father, past experiences). Again, several of this are included in the manuscript, but these could be organized more clearly. It would be also upon of the reorganization of the manuscript (which is the main point to improve).

Unfortunately, there is no clearly defined body of theory to explain the process of choosing the capital city. The various pieces of information are incomplete and do not always coincide. We have chosen to show a realistic, though perhaps confusing, view of the state of knowledge. We have reorganized the manuscript and believe it is now easier to follow.

10. In page 44, it is mentioned that “according to the naïve map, the city which best met the requirements to be the capital was Madrid, followed by Valladolid, Murcia, and Barcelona”.

a. From, figure 12, it appears that whilst Madrid was first, second was Murcia, and almost equally Valladolid and Barcelona. Please check it.

Thanks for the appreciation. The three follower cities were identified by the order in which they appear in the graphic and that coincides with their position in the ranking of the real map. In the new version they appear by their position in the naive map ranking.

b. Also, the difference seems to be very minimal. The authors could clarify how this small difference could have influenced the decision at that time (when similar methods and data employed by the authors were not available).

The results do not show great differences. As has been pointed out in the new version of the text, the criteria applied rather than determining the specific city that should have been chosen indicate the small group of cities that enjoyed the right conditions to be the capital of the Hispanic Monarchy. The final choice would depend on other economic, political or even personal conditions of the king.

The data used for the analyses are available in an online repository.

11. The conclusion could be

Some modifications were introduced in the conclusions section together with some considerations on the possible implications of the paper for future work.

12. Internal references need to be revised

a. For example, in page 32, the reference is to Figure 2, please confirm if that is the adequate.
b. Similarly occurs with the reference to Figure 4 in page 38

Thanks for the indications. The errors in the figure references have been corrected.

13. The manuscript is related to previous work from the authors. This is acknowledged, but from a personal point of view, it could be explicitly mentioned as “in a previous study, the authors…” (or similar sentence). That is a matter of personal preference, so I have no strong opinion on it.

We have modified the text following your suggestion.

Thank you for your comment and the rest of your suggestions and indications. We believe that they have been very useful to improve the deficiencies observed in the structure of the text.

**Reviewer #2**

This paper aims at revisiting the choice of Madrid by Felipe II de España as the capital based on limited knowledge at the time, especially in terms of the economic validity. It gives us not only deeper understanding of the Spanish land development history but also interesting insights and argument for the future development. Some further verification would however be necessary to have good grounds for estimating the economic soundness of Madrid as the capital. Moreover, I'm afraid that some parts of the article might be difficult to follow and the English writings could be improved. Overall, I'd recommend this paper for the journal for its unique findings of academic and practical importance with additional validation of the conclusion and careful editing of the English writings.

Thank you for your kind and constructive comments. We have made a thorough revision of the text which we hope you will find satisfactory.

***(1) Further validation of the conclusion***

If I understand correctly, the choice of Felipe II is assumed to be based on limited knowledge, namely, the proposed naïve map in this research. It would be true that Madrid had a good reason to be chosen as the capital for its economic advantages, even with limited information (the "naïve map"), and that choice could be assessed as economically rational with the present knowledge (based on the "real map"). But I don't see if the gaps of the index scores between Madrid and other options, especially Valladolid, Murcia and Barcelona, are so significant that we can be very confident that Felipe II took the economic validity into consideration in the choice of capital. I'd suggest that further examination of data and argument be made to elaborate the evaluation of the 'candidate' cities based on your network analysis so that you can conclude Madrid was "clearly (...) the best option" (p.46).

Valladolid and Madrid had relative advantages in (then perceived) economy, like Murcia and Barcelona, based on your network analysis. And Madrid would have been more competitive in terms of the intrinsic characteristics. Madrid also had the predominance of urban and administrative activities and the high concentration of nobility, which can justify the choice perhaps politically, practically and financially. But again, it would not be easy to conclude Madrid was the best choice solely from the results of the network analysis.

Thank you for your comments. In the new wording of the text, the expressions have been softened to indicate that the choice was reasonable rather than optimal.

***(2) Polishing the English writings***

There would be many sentences in the paper that are not easy to follow. (For instance, "However, it looks strange for Madrid's suitability from an economic perspective not to have been considered by Philip II and his advisers. However, despite the importance of the issue, it has attracted little direct analysis" (p.6). The first sentence could be differently written for easier understanding/ The repetition of 'however' should be avoided. In the same page (p.6), another sentence which requires some improvement would be "According to such suggestions, Madrid's main advantage, from Philip II's point of view, was probably that neither the Church nor the nobility enjoyed an important presence there, which would facilitate the development of a new administration, under the solely under royal supervision.")

Also, many repeated contents are found; for instance, the conclusion section has too many repeated sentences of the previous section. Such simple repetition should be removed, and your thorough editing, or restructuring, of the paper would also be recommended to make the paper more efficiently written, that is, reader-friendly. A proofreading by native English speaker, preferably a professional, should be performed.

We apologize for the hasty drafting of the text. A thorough revision of the text has been carried out. We hope that the new version will be easier to read.

***(3) Possible further discussion on the findings***

Among the four major options for the capital (Madrid, Valladolid, Murcia and Barcelona), especially from the restricted assessment at that time (the "naïve" map), only Barcelona seems to have a rather big gap between the assessment based on the "real" map and that based on the "naïve" one. In what way can we interpret its relatively low evaluation based on the "real" map, compared to the subsequent economic and urban development? If your interpretation on this issue about Barcelona affects the conclusion on Madrid in some way, it could be added to your paper.

Although a more detailed explanation would require a specific analysis. The reason for the better results of Barcelona (and Zaragoza) in terms of the naive analysis than the real one lies in the variation of the center of the road network towards the northeast. As can be seen in Figure 13, with the 16th century view Barcelona appeared to have better accessibility than the real one. A brief reference to this has been included in the text.

***(4) Minor issues***

- p.4: "Thus, his sister, Juana of Austria, regent between 1554 and 1556, and between 1556 and 1559, she told him in August 1958 that she preferred Madrid to Valladolid as the capital, or, failing that, Guadalajara or Burgos". -> It should be August 1558, not 1958.
- p.5: "Therefore, the establishment of a permanent capital meant the renunciation of the Carolinian practice of the itinerant monarchy, a course which, with all its drawbacks, had the great advantage of occasionally giving its peoples visible proof that their king had not forgotten them". -> It should be people, not peoples.
- p.6: "However, this does not mean that it has not been commented on and discussed a great deal, with religious and political reasons often cited as to why Madrid was more suitable than, say, Valladolid Toledo, Seville, or even Barcelona or Lisbon". -> "," is missing between "Valladolid" and "Toledo".
- p.7: ""The very existence of Madrid as a capital city was the consequence of a political decision. No other city in early modern Europe was as dependent upon administered economic life, and no major city was so poorly located to stimulate market-oriented exchanges" (Ringrose 1983, p. 8)". -> "Europe" should be in italic.
- p.8: "Madrazo Madrazo (1984b) attempted to identify the infrastructure work carried out under the Bourbons". -> In some parts, authors are referred with its first and second surnames, while they have only the first surname in others. Better to be coherent?
- p.9: "According to data from Bairoch et al. (1988), at the beginning of the 16th century, the most populated cities in Spain were located in the south (Seville and Malaga) and in the east (Valencia), all three having more than 40.00 inhabitants". -> It should be 40,000, not 40.00. Also, punctuation of three digits (thousand) should be coherent throughout the paper, preferably comma in English.
- p.11: "It did not have enough houses and its location on a hillock and surrounded by the River Tajo made it difficult to build more. r(Alvar Ezquerra 1985)" -> There is an unnecessary "r". And, the reference information should be before the period.
- p.11: "Although it may seem strange to us now, the exposition to epidemics was a question of crucial importance, mainly talking about the capital location where the high number of travelers passing through was an element of added risk". -> "(E)xposition" should be exposure, I guess. And, there are too many short paragraphs in this page.
- p.18: "3. The logistical advantages of the port cities depend on the latency of the network and the goods transported". -> "3" should be in black color.

- **** I don't raise all those points to be revised. There seem to be a lot... Please have a thorough proofreading.**

- P.20: I don't well understand what "Estimated year data" means in Fig. 3.
- P.35: "The review of the somewhat disjointed information available confirms that the Villuga and Meneses itineraries, although useful, do not offer a reliable picture of the characteristics of the sixteenth-century road network". -> What is "somewhat disjointed information"? A bit more careful expression would be appreciated.

Thank you for your detailed comments. We hope we have resolved the errors in the text.
