# Supplementary material for: The choice of Madrid as the capital of Spain by Philip II in the light of the knowledge of his time: A transport network perspective
Source: PLoS One. 2022 Jun 15;17(6):e0269419. doi: 10.1371/journal.pone.0269419 (PMC9200313; doi:10.1371/journal.pone.0269419)
Supplement: Attachment — Submitted filename: Response to Reviewers.docx [file pone.0269419.s005.docx]

Rebuttal letter

*Thank you again for your kind and thoughtful comments. We have tried to respond to them adequately.*

# Reviewer #1: (No Response)

# Reviewer #2:

Your thorough revision responding to our comments is highly appreciated. I see huge improvements, especially in the framing of your study (objectives and conclusions) which now matches better your data and analysis. However, there remain two major issues to be addressed in your paper.

*Following your suggestion we have enlisted the help of a company specialized in editing scientific papers. We hope that the text will now be more understandable.*

(1) The quality of English
I'd say that the quality of English is not enough yet for recommending to an international journal with readers from all over the world. There are a lot of sentences with a nest of too many clauses/phrases. Readers would find it very difficult to follow. Particularly, the first and second chapters ("Introduction" and "Advantages and disadvantages of candidate cities considered on their own") should be re-edited. It is highly recommended that you get advice from native English speaker, not only about grammar but also about reader friendly writing.
(An example in p.4: "Elliot (10) also pointed out that Philip II was mistaken in believing that the decision to reside in the geometrical center of the peninsula would produce the impression of absolute impartiality in the treatment accorded to his subjects, since, although this was not the intention of Philip II, the very choice of the capital in the heart of Castile granted his government a Castilian color".)
(Another example in p.8: "From the point of view of urban functionality, the narrow streets of Toledo, still organized along medieval lines, did not allow the court to move with adequate splendor and ease, making it difficult to ride on horseback through many of the streets and preventing movement by carriage along most of them (44)".)

In addition, there remain many typographical errors and some grammar issues.
Please look at my comments on the attached manuscript.
Without seeing a significant improvement in writing, especially error correction, it would be difficult for me to recommend your paper to the journal. Again, your very careful proofreading would be strongly advised.

(2) The position/meaning of the analysis of intrinsic characteristics in the whole structure
As your abstract and conclusion imply, the network analysis with using the two maps (one of your novel contributions) would be primary and the analysis of intrinsic characteristics would be supplementary. The position of the second chapter ("Advantages and disadvantages of candidate cities considered on their own") would thus look unclear in the whole structure. It can perhaps be discussed after the network analysis.
If you have any clear reason to put this chapter here (right after the introduction and before the network analysis), the logical flow between this chapter and the next should be improved. The structure of the conclusion could also be reconsidered.

*Regarding the comments on the structure, we have made some small changes but we have kept the general outline. The objective has been to maintain the habitual scheme in the works on the capital of Madrid. The unusual length and structure of the article is due to the need to justify in great detail the assertions made about the advantages of road transport as they represent a very different view from the current one. We believe that with the improvements introduced during the editing process the work will be of interest to a wide audience.*

*The conclusions have been expanded and reordered.*
